# Double burden: a cross-sectional survey assessing factors associated with underweight and overweight status in Danang, Vietnam

**DOI:** 10.1186/1471-2458-13-35

**Published:** 2013-01-14

**Authors:** Kiet A Ly, Thanh GN Ton, Quang V Ngo, Tung T Vo, Annette L Fitzpatrick

**Affiliations:** 1 Department of Oral Health Sciences, School of Dentistry, University of Washington, Seattle, WA, USA; 2 Department of Neurology, School of Medicine, University of Washington, Seattle, WA, USA; 3 Department of Health, Danang, Vietnam; 4 Departments of Epidemiology & of Global Health, University of Washington, Seattle, WA, USA

## Abstract

**Background:**

Many low- to middle-income countries are faced with an increasing prevalence of overweight/obesity while that for underweight remains high, a duality termed “double burden”; both are key risk factors for chronic diseases. This cross-sectional study assesses the prevalence and factors for underweight and overweight/obesity among adults in Danang, Vietnam, using WHO standard and suggested Asian-specific BMI cut-offs.

**Methods:**

In 2010, 1713 residents age ≥35 years from 900 households in 6 of 56 urban, rural and mixed urban–rural communes in Danang were selected using multistage-cluster sampling methodology to participate; 1621 qualified adults enrolled. Participants completed a health survey based on WHO STEPwise Approach to Chronic Disease Risk Factor Surveillance and additional questions on chest pain and stroke symptoms. Anthropometric and other measurements were conducted. Relative risk regression was used to identify independent risk factors for underweight or overweight/obesity according to WHO standard cut-offs and suggested Asian-specific cut-offs (<18.5 kg/m^2^ or 23–27.49 kg/m^2^; and ≥27.5 kg/m^2^).

**Results:**

We observed 12.4% prevalence of underweight and 16.0% for overweight/obesity using WHO standard. The prevalence of overweight/obesity doubled (33.7%) when Asian-specific cut-offs were applied. For both definitions, rural communes had the highest prevalence of underweight while urban communes had the highest prevalence of overweight/obesity. Being underweight was associated with less urbanization. Factors independently associated with being underweight included older age, rural living, current smoking, and lower systolic pressure. Factors independently associated with Asian-specific BMI definition for being overweight/obese included older age, urbanization, higher systolic pressure, and diabetes. Age was not an independent factor with WHO standard cut-offs; however, myocarial infarction and diabetes showed strong associations.

**Conclusions:**

The double burden of underweight and overweight/obesity observed in Danang is consistent with patterns found for large cities in Vietnam that are undergoing rapid economic growth and urbanization of lifestyle. Factors independently associated with underweight and overweight/obesity status by WHO standard and Asian-specific definitions include urbanization and modifiable lifestyle factors. Further studies are needed to define ethnic specific BMI cut-offs for Vietnam and to explore strategies to reduce the rising prevalence of overweight/obesity.

## Background

Overweight and obesity are recognized as global public health problems because they are associated with increased risk of many diseases including hypertension, type 2 diabetes, cardiovascular diseases, stroke, osteoarthritis, and some cancers [1,2]. Similarly, underweight is a risk factor for many chronic diseases such as respiratory illnesses, osteoporosis, as well as diabetes, hypertension, and cardiovascular diseases [3-5]. With continuing economic growth over the last two decades, many low-to-middle income countries in Asia are facing increasing prevalence of overweight/obesity while underweight remains a public health challenge. The dual burden of underweight and overweight/obesity has been termed the “double burden” of diseases [6], and has received considerable attention in Asia [3,5,7,8] where rapid economic growth and development, urbanization, and associated change in dietary and lifestyle patterns are fueling the rapid rise of overweight/obesity and diet-related diseases.

The World Health Organization’s (WHO) “standard” definition for underweight, overweight, and obesity by body mass index (BMI) are ≤ 18.5 kg/m^2^, 25 to <30 kg/m^2^, and ≥ 30 kg/m^2^, respectively [9]. However, Asian populations appear to have greater cardiovascular risk, higher morbidity and all-cause mortality than western populations at any given BMI level [10].

Currently debated is whether the standard WHO BMI cut-off points for overweight and obesity are appropriate for use across racial and ethnic groups, particularly among Asians. Differences in lifestyle factors, limbs, trunk, and stature proportionality, and in ethnic variations of body fat distribution in relation to BMI have been debated [11]. Similarly, waist circumference, another indicator for overweight and obesity, is also facing debate regarding appropriate cut-off points for different racial ethnic groups. In a large prospective study with 5,515 Europid and 2,214 ethnically South Asians, Cameron and collegues [12] found that using the current recommended waist circumference cut-offs, South Asian men and women had significantly higher estimated incidence of diabetes (5.8% and 2.1%, respecitively) compared to their Europid counterparts (0.6% and 0.4%, respectively). Studies attempting to define waist circumference cut-offs as related to ethnic Asians are numerous [13-17] and growing. The WHO Expert Consultation Report of 2004 acknowledged the existence of greater risk at lower BMI for Asians and that Asian-specific BMI cut-off points for better assessment of risk-related diseases may be needed. The panel suggested retaining the standard WHO BMI cut-offs as the international classification but identified further “potential public health action points” at 23.0, 27.5, 32.5, and 37.5 kg/m^2^ along the continuum of BMI [18].

Numerous studies conducted among various Asian ethnic groups have attempted to justify lower BMI cut-offs for assessing overweight-related disorders and for evaluating obesity-related morbidities and mortalities [10]. While further work is needed to define the appropriate Asian- or ethnic Asian-specific BMI cut-off points, what is clear is the existence of the “double burden” of underweight and overweight/obesity and their associated increased morbidity and mortalities in low- and middle- income countries. In Vietnam, Ha and colleagues compared National nutrition data collected in 2000 and 2005 and reported that Vietnam was experiencing this double burden with a trend towards decreasing underweight and increasing overweight/obesity over the two time points [19]. Other studies conducted in major economic regions in northern and southern Vietnam reported even greater prevalence of overweight and obesity compared to national findings [5,20,21]. Danang City is a major economic hub in central Vietnam and, to date, a study evalutating weight status of its population has not been reported.

Our goals were to (1) estimate the prevalence of underweight and overweight/obesity in a population-based sample of adults age 35 years and older living in Danang City; (2) identify independent factors associated with being underweight and overweight/obese by two definitions, the standard WHO and the WHO suggested Asian-specific BMI cut-off points; and (3) compare our findings with those previously reported for Vietnam.

## Methods

### Setting

Danang City is located on the southern Central Coast of Vietnam covering approximately 1,255 square kilometers with a population of 890,500 residents. According to the Vietnam General Statistics Office, Danang City is one of five municipalities that are administratively equivalent to provinces and directly under the central government similar to Hanoi, HoChiMinh City, Haiphong, and Cantho city. Danang City is the fourth most populated city and is one of three major port cities. Its urban growth rate was 3.5% with the highest urbanization ratio (87%) among Vietnam’s cities and provinces [22]. Danang City GDP growth rate averaged 11% from 2006–2010, 1.5 times the national average. Danang City is also the economic and academic hub of Central Vietnam [23]. Administratively, Danang City is subdivided into districts, which contain either wards or communes. These are further subdivided into hamlets or villages. For convenience, this paper will use “communes” for communes and wards, and “hamlets” for hamlets and villages.

### Study design

This is a cross-sectional survey that uses a stratified multistage cluster sampling technique to identify households for inclusion in this study. The Danang People’s Committee provided a list containing the Danang City administrative units and household statistics. The 56 communes in Danang City were stratified into urban, mixed urban/rural, and rural as determined by local government officials - the heads of each local Commune Health Clinic. Mixed urban/rural communes were defined as those that contain rural areas covering 30% to 50% of their geographic boundary. Of the 56 communes in Danang City, 66% are urban, 23% are rural; and the remaining 10% are mixed urban/rural.

We carried out a three-stage cluster sampling stratified by urbanization. We also used disproportionate sampling to allow for sufficient between-strata estimation while minimizing cost and maximizing operational efficiency for data collection [24]. The primary sampling units were the 56 wards. The secondary sampling units were hamlets in the selected wards. The last sampling units were households in the selected hamlets. All individuals ≥35 years of age residing in the selected households were invited to participate in the study. In each strata, we randomly selected communes: 3/37 urban, 2/13 rural, 1/6 mixed. Second, we randomly selected 5 hamlets within each chosen commune (30/242 total hamlets). Third, we randomly selected 30 households within each chosen hamlets (900/3241) total households. The University of Washington’s Institutional Review Board approved all study procedures. An equivalent division in the Danang Department of Health also reviewed and approved all study protocol. All study participants provided informed consent.

### Data collection

Study trained commune health staff recruited participants from selected households, and made appointments for participants to visit the local commune health center. During the visit, an interviewer-administered survey was completed. The WHO STEPS instrument from the WHO STEPwise Approach to Chronic Disease Risk Factor Surveillance was utilized as the basis for survey [25] which included questions on demographics, tobacco and alcohol use, physical activity, self-reported history of disease, and dietary intake. Six questions on stroke symptoms from the Questionnaire for Verifying Stroke-Free Status (QVSFS) [26] were also included. Questions on chest pain were from the Rose Angina Questionnaire [27]. These questions were further supplemented by questions on physical function, anxiety and stress taken from other validated instruments.

The clinical assessment included a series of three resting systolic and diastolic blood pressure (mmHg) and heart rate (beats/minute) measured using a digital blood pressure device (Microlife USA, Inc., Clearwater, FL). Participants were asked to sit and relax for 5 minutes prior to blood pressure measurement. Weight (± 0.2 pounds) and percent body fat (± 0.1%) were measured using the OMRON Model HBF-400 scale (Omron Corporation, Kyoto, Japan). Height was measured using a standard measuring tape and a head level. Participants were asked to remove their shoes, hat, belt, and pocket items before weight and height measurements were conducted. Spirometry was repeated three times using a Microlife, PF100 digital peak flow meter (Microlife USA, Inc., Clearwater, FL, USA) to obtain forced expiratory volume (FEV) and peak expiratory volume (PEV). Balance and gait were carried out using components in the Short Physical Performance Battery [28].

Two health care workers stationed at each commune health clinic collected the data. Centralized training for data collectors was held to review study procedures, make final modifications, pre-test forms and survey instruments, and conduct field exercises of study procedures. Data were collected between July and December 2010. Study physicians visited the study sites periodically throughout the study period to monitor quality and assure standardization of procedures.

### Statistical analysis

BMI was calculated and categorized using (1) the WHO “standard” cut-offs, as follows: underweight (< 18.5 kg/m^2^), overweight (25 – 29.99 kg/m^2^) and obese (≥ 30 kg/m^2^), and (2) the Asian-specific cut-offs: underweight (< 18.5 kg/m^2^); overweight (23 – 27.49 kg/m^2^); and obese (≥ 27.5 kg/m^2^) [8]. Based on the administrative lists, we calculated sampling weights for each stage of clusters sampled, and subsequently assigned a final sampling weight as the product of weights at each stage. Using statistical survey methods to re-weight our sample back to the population [29], we calculated the prevalence of underweight and overweight/obesity status using two BMI definitions. We conducted separate univariate analyses to assess factors associated with being overweight and underweight/obese. We conducted our analyses for standard WHO cutoffs and repeated for cut-offs suggested for Asian populations. In univariate analyses for categorical variables, we conducted Pearson chi-squared tests corrected for survey design with the second-order of Rao and Scott [30], and converted the test into a design-based F statistic. To test the difference in means of continuous variables across body weight categories, we used generalized linear regression to obtain the Wald test adjusted for the clustered survey design [31]. To test for linear trend of ordered categories, we modeled the ordered categories as one continuous exposure variable and conducted an adjusted Wald test. We conducted multivariate analyses to identify independent factors associated with being underweight and being overweight/obese in separate models. Because neither underweight nor overweight/obese status were rare events in our sample (i.e., > 10%) and because odds ratios provide biased estimates of prevalence ratios when outcomes are common [32], we used generalized linear models instead of logistic regression to obtain prevalence ratios associated with risk factors and demographic and clinical characteristics [33]. We assumed Poisson distribution for the variance to fit our models. All multivariate analyses accounted for the multistage cluster design using survey estimation methods in Stata 11.2 (College Station, TX). We identified independent factors associated with underweight and overweight/obesity status in regression models using a backward stepwise approach. All factors that were significantly associated with BMI categories in univariate analyses were placed into the full model and removed sequentially if corresponding P-value of the adjusted Wald test exceeded 0.05. Variables selected for potential inclusion in the model were age, gender, income, education, urbanization, smoking status, number of drinks/week consumed, number of days eating fruits/vegetables per week, measured systolic and diastolic blood pressure (per 10 mm/Hg), low physical activity (defined by WHO STEPS survey), self-reported history of stroke, number of stroke symptoms, diabetes, high cholesterol, severe chest pain lasting 30 minutes or longer, cancer, forced expiratory volume, peak flow, and timed walk. Factors that were significantly and independently associated with each outcome were retained in the final models. Because the WHO also suggests Asian-specific BMI cut-offs for “potential public health action points”, we repeated our analyses using the suggested cut-offs: < 18.5 kg/m^2^, 18.5 – 22.99 kg/m^2^, and ≥ 23 kg/m^2^ for underweight, normal, and combined overweight/obese, respectively. All tests were two-sided and statistical significance was defined as P-value < 0.05. Analyses were performed in Stata version 11.2 (College Station, TX).

## Results

**Figure 1:**
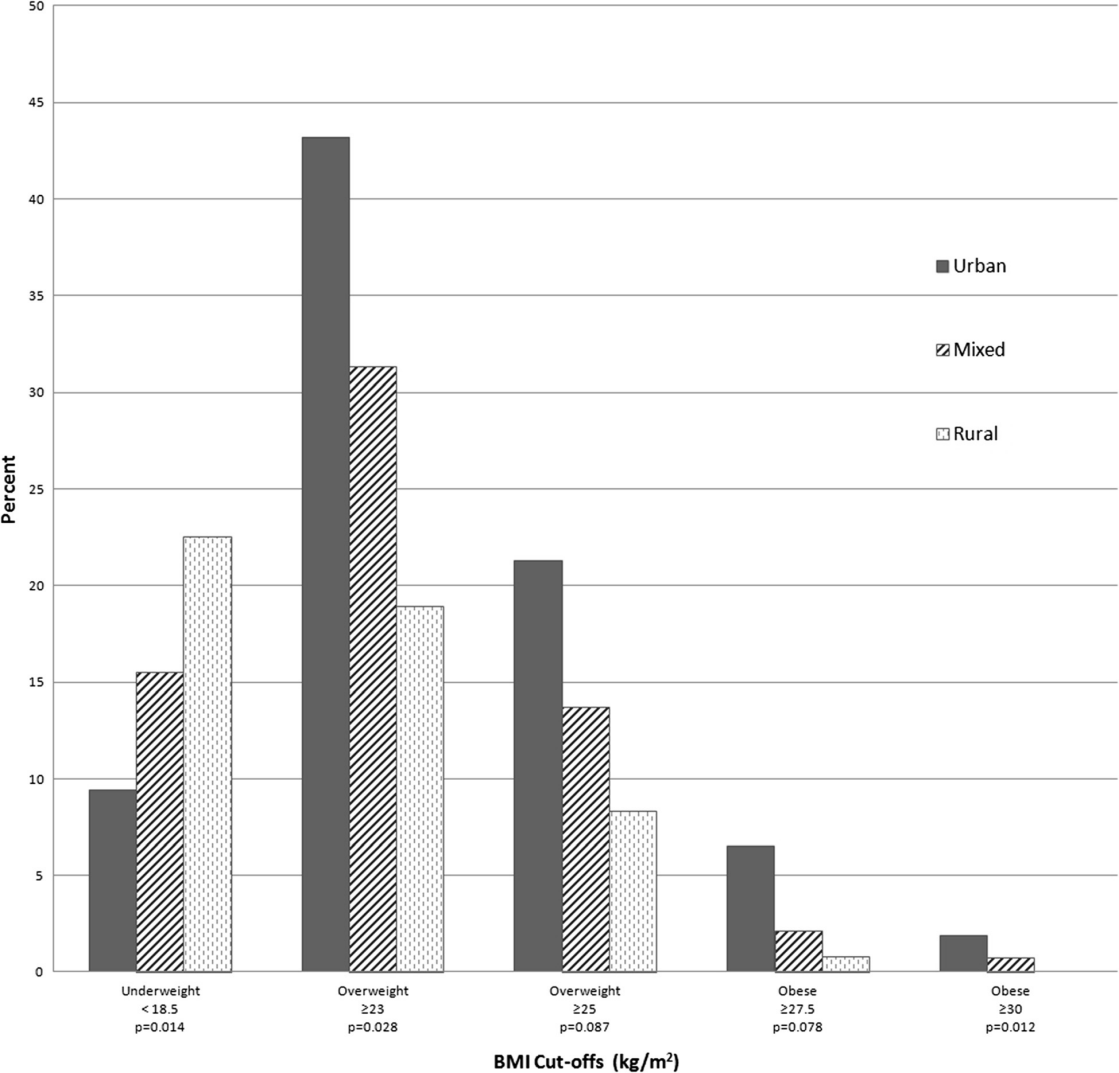
Prevalence of Underweight, Overweight, and Obese Status Among Adults 35–93 Years Old According to Urbanization in Danang City, Vietnam, 2010.

**Figure 2:**
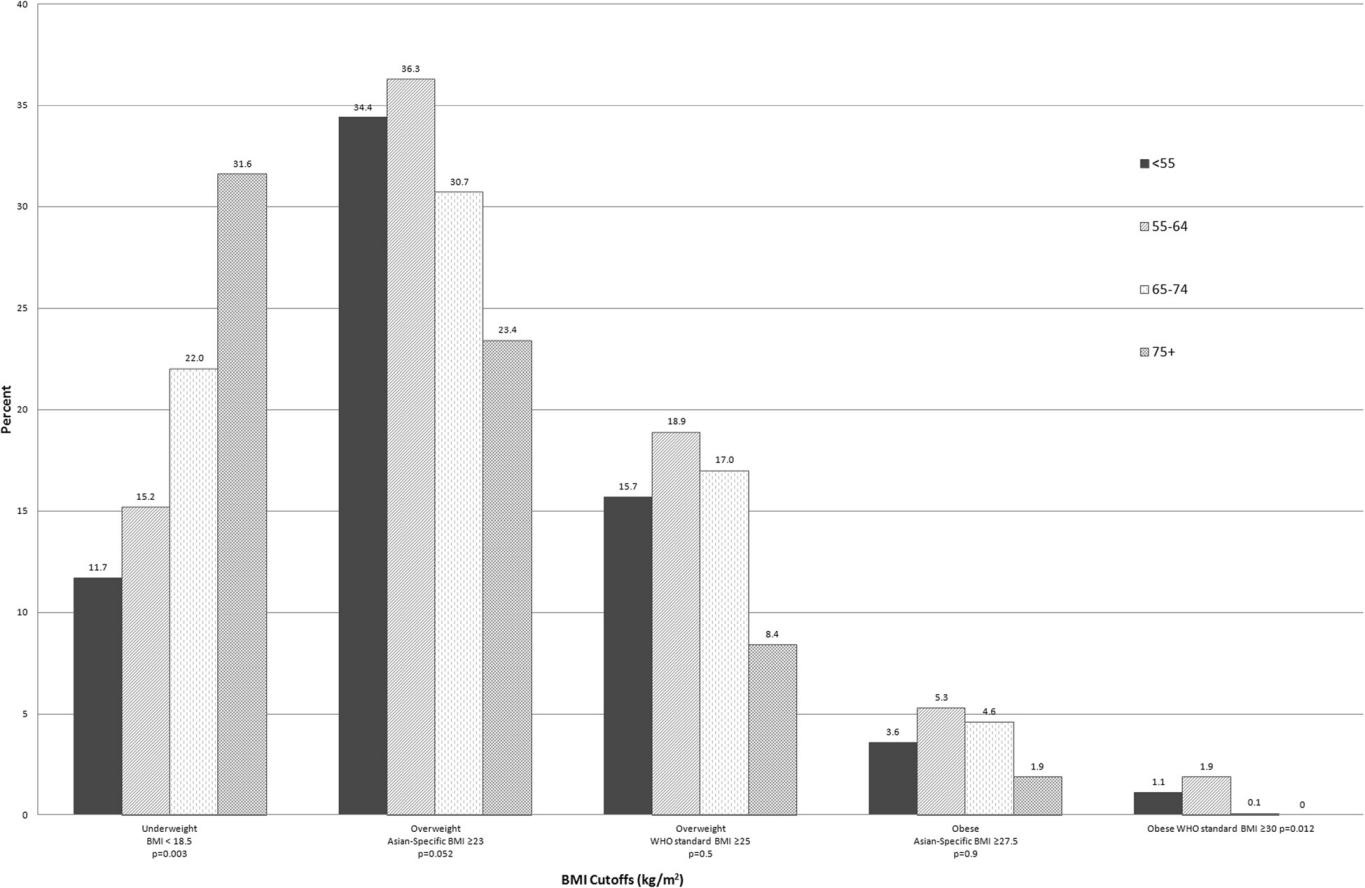
Prevalence of Underweight, Overweight, and Obese Status Among Adults 35–93 Years Old According to Age in Danang City, Vietnam, 2010.

Of the 1,713 adults approached for inclusion in the study, 92 from 17 households declined participation resulting in a response rate of 94.5%. A total of 1,621 adults from 887 households provided informed consent and completed the study. Age of participants ranged from 35 to 93 years with a mean of 52.0 years (± 12.5); 43.9% were men. The majority (64.1%) were age 35 to <45, and 6.7% were >75 years of age (Table 1). The most commonly self-reported condition was tooth pain (31.2%) followed by joint pain (23.1%), gum pain (14.1%), and hypertension (14.1%). Of note, measured hypertension (SBP>140 or DBP>90) showed nearly twice (27.7%) the prevalence of self-reported hypertension. Over 25% of participants were current smokers and another 14.4% were former smokers, almost all of whom were men. The overall prevalence for underweight and overweight/obesity was 12.4% and 16.0%, respectively, using the WHO standard cutoff of BMI ≥ 25 kg/m^2^. When we applied the suggested Asian-specific overweight/obesity cutoff of BMI ≥ 23 kg//m^2^, the prevalence doubled (33.8%). Patterns in prevalence of weight status were evident across communes with varying degrees of urbanization. Prevalence of underweight status rose from 7.4% to 13.4% to 20.5% across urban, mixed urban/rural, and rural communes, respectively – that is, the prevalence increased with decreasing degree of urbanization (p < 0.014, test for trend). Regardless of which BMI cut-offs were used to define overweight and obesity, prevalence lowered significantly with decreasing levels of urbanization (Figure 1). The highest prevalence of overweight status occurred in urban communes for both definitions of overweight/obese status, reaching 43.2% with Asian-specific BMI ≥ 23 kg/m^2^, Figure 2. The prevalence of underweight status generally rose with older age. For either WHO standard and Asian-specific BMI definitions, no linear trends in the prevalence of overweight/obesity were evident according to age; however, the prevalence appeared the highest among 55 to 64 year olds, and lowest among those 75+ years (Figure 2).

**Table 1:** Prevalence of Body Weight Status According to Demographic, Reported History, and Measured Variables Among Residents 35 to 93 Years in Danang City, Vietnam, 2010

	**Total**	**Standard WHO Cutoffs**				**Suggested Asian Cutoffs**		
		**Underweight**	**Normal**	**Overweight or Obese**	P-value*	**Normal**	**Overweight or Obese**	P-value*
Characteristic		<18.5 kg/m^2^	18.5 – 24.9 kg/m^2^	≥ 25 kg/m^2^		18.5 – <23 kg/m^2^	≥ 23 kg/m^2^	
Participants, n**	1621	200	1151	258		866	543	
Male, %	43.9	39.5	44.7	44.2	0.17	45.6	44.0	0.17
Age, years, %					0.013			0.0031
<45	64.1	51.0	66.6	63.0		66.2	65.4	
45-64	19.7	19.5	19.0	23.4		18.8	21.3	
65-74	9.5	14.0	8.6	10.1		9.0	8.7	
75+	6.7	15.5	5.8	3.5		5.9	4.6	
Age, years, mean	52.0	56.7	51.0	52.1	0.031	51.1	51.4	0.017
Urbanization, %					0.033			0.010
Urban	52.0	31.0	51.8	69.0		47.7	66.5	
Mixed urban–rural	18.1	19.5	18.4	15.5		18.6	16.8	
Rural	29.9	49.5	29.8	15.5		33.7	16.8	
Monthly income ^†^, %					0.013			0.016
<2 million dong	20.1	37.4	18.7	13.3		21.2	20.1	
2-3.9 million dong	31.8	34.4	32.0	28.9		34.0	31.8	
4-5.9 million dong	21.8	16.9	22.2	23.8		20.4	21.8	
>6 million dong	26.3	11.3	27.1	34.0		24.4	26.3	
Education ^†^, n(%)					0.089			0.029
No formal schooling	4.9	10.6	4.2	3.5		4.4	3.5	
Grade school	78.0	81.5	78.0	75.2		79.6	74.2	
High school	6.9	5.0	7.3	6.6		7.2	7.2	
University or post-grad	10.3	3.0	10.5	14.7		8.9	15.1	
Reported history ^†^**,** %								
Tooth pain	31.2	30.0	32.6	26.0	0.2	30.8	32.2	0.7
Joint pain	23.1	24.0	22.4	25.2	0.7	22.1	24.3	0.7
Gum pain	14.1	13.5	13.2	14.0	1.0	12.8	16.2	0.4
Hypertension	14.1	10.5	12.8	22.9	0.036	11.5	19.7	0.022
High cholesterol	6.0	2.0	4.8	14.7	0.008	3.7	11.2	0.013
Diabetes	4.0	2.0	10.2	4.0	0.014	2.2	7.5	<0.001
Chest pain ≥ 30 min	4.8	4.0	4.8	5.4	0.8	4.3	5.9	0.5
Myocardial infarction	0.2	0.0	0.1	1.2	0.072	0.1	0.5	0.2
Stroke	0.2	0.0	0.2	0.4	0.7	0.2	0.2	0.8
No. stroke symptoms ^†^, n%					0.18			0.2
0	75.5	69.4	76.0	78.0		75.5	77.8	
1	12.2	12.1	12.0	13.3		11.8	13.0	
2	8.3	10.6	8.5	5.5		9.4	5.8	
3+	3.9	8.0	3.4	3.1		3.4	3.3	
Smoking status ^†^, %					0.065			0.064
Nonsmoker	59.3	49.0	60.1	64.0		57.7	65.8	
Former smoker	14.4	13.0	13.9	17.8		14.7	14.6	
Current smoker	26.2	38.0	26.0	18.2		27.6	19.7	
Drinks per week^†^, %					0.2			0.4
None	65.2	71.5	64.9	61.6		64.2	64.5	
< ½	14.1	8.5	14.2	18.2		14.5	15.7	
½ to 2	9.4	10.5	9.1	9.7		9.0	9.6	
≥ 3	11.3	9.5	11.8	10.5		12.3	10.3	
Clinical Measurements								
Systolic, mmHg, mean	128.2	123.7	127.8	132.9	0.031	127.5	130.6	0.02
Diastolic, mmHg, mean	80.6	77.3	80.3	84.5	0.018	80.0	82.8	0.016
Hypertension (SBP ≥ 140 or DBP ≥ 90)	27.7	19.5	26.9	38.0	0.047	26.3	33.0	0.004
Fast walk, seconds, mean	3.6	4.1	3.6	3.4	0.4	3.6	3.4	0.4
FEV, L/min, mean	2.0	1.89	2.02	2.02	0.02	2.01	2.03	0.02
PEF, L/min, mean	2.1	2.0	2.1	2.1	0.2	2.1	2.1	0.2

*p-value for design-based F statistic.

**Population prevalence estimates based on reweighting for these participants in sample.

^†^ Self-reported.

When stratified according to mutually exclusive groups of WHO standard body weight status for underweight, normal, and overweight/obese, we observed univariate associations (p < 0.05) with age, urbanization, household income, self-reported history of hypertension, diabetes and high cholesterol, measured systolic and diastolic blood pressure, measured hypertension, and FEV (Table 1). Associations were not observed for sex, education, or smoking. Similar factors were significantly associated with weight status when we used the Asian-specific cut-offs except for education, which became statistically significant.

In our multivariate models, factors that were independently associated with higher prevalence of underweight status included older age, less urbanization, current smoking status, and lower systolic blood pressure (Table 2). Older age was associated with a higher prevalence of underweight status with the oldest residents (75+ years) having nearly 3-fold higher prevalence than those younger than 55 years of age. Current smokers had the highest prevalence of being underweight relative to never-smokers, with a significant increasing trend evident across the categories for non-smokers, former smokers, and current smokers (p = 0.028). Finally, lower systolic pressure was associated with higher prevalence of being underweight with each 10 mmHg increase associated with a 12% lower prevalence of being underweight after adjusting for all other factors.

**Table 2:** Independent Factors Associated with Underweight and Overweight Status Among Residents 35 – 93 Years of Age: Danang Province, Vietnam, 2010

	Underweight < 18.5*			**Overweight as BMI ≥ 23**			**Overweight as BMI ≥ 25**		
	**Prevalence Ratio**	**95% CI**	**p-value**	**Prevalence Ratio**	**95% CI**		**Prevalence Ratio**	**95% CI**	**p-value**
Age^‡^			0.001			0.047			
<45	1.00	Reference		1.00	Reference		--	--	
45-64	1.47	(0.88, 2.47)		0.96	(0.78, 1.19)		--	--	
65-74	2.11	(1.47, 3.03)		0.87	(0.60, 1.28)		--	--	
75+	2.76	(2.26, 3.38)		0.79	(0.62, 1.00)		--	--	
Urbanization			0.029			0.045			0.13
Urban	1.00	Reference		1.00	Reference		1.00	Reference	
Mixed urban–rural	1.55	(0.91, 2.64)		0.78	(0.60, 1.01)		0.71	(0.50, 1.00)	
Rural	2.12	(0.98, 3.47)		0.52	(0.27, 1.00)		0.47	(0.13, 1.74)	
Smoking Status^‡^			0.028						
Never	1.00	Reference		--	--		--	--	
Former	1.11	(0.41, 2.41)		--	--		--	--	
Current	1.80	(1.13 2.16)		--	--		--	--	
Systolic pressure (10 mmHg)	0.88	(0.77, 0.99)		1.06	(1.03, 1.12)		1.11	(1.00, 3.72)	
Diabetes^†^	--	--		1.60	(1.37, 1.86)		2.12	(1.20, 1.45)	
Heart attack^†^	--	--		--	--		4.67	(1.37, 15.9)	

*underweight defined as BMI<18.5; reference group defined as normal BMI (18.5-24.9); 258 subjects overweight (≥25) were excluded from analyses.

^†^Adjusted for all other factors in table.

Independent factors associated with being overweight/obese by standard WHO and Asian-specific BMI definitions are presented in Table 2. Older age was significantly associated with a lower prevalence of being overweight/obese by the Asian-specific definition but not by the WHO standard definition. Those from rural communes were less likely to be overweight/obese by both definitions. Unlike the model for underweight status, smoking status was not an important independent factor for being overweight/obese by either definition. The associations for systolic blood pressure and diabetes were stronger in the Asian-specific definition. The association between self-reported heart attacks and being overweight/obese was notably strong for the model using the standard WHO cutoff (prevalence ratio = 4.67; 95% CI; 1.37, 15.9).

## Discussion

Results of this study provides evidence of the double burden of underweight and overweight/obesity experienced in Danang City, Vietnam. Our 12.3% overall prevalence of underweight status was similar to the 12.6% reported by Walls and colleagues [5] for their study conducted in 2004 among provinces surrounding Hanoi, where the population characteristics (i.e. urban, mixed urban–rural, and rural) and economic development were similar to our population. On the other hand, our underweight prevalence was much lower than the 20.4% for the 2004 urban Ho Chi Minh City (HCMC) cohort [21] and 20.9% reported by Ha and colleagues for the 2005 Vietnam National Adult Obesity Survey (VNAOS) [19]. The comparision needs to take into consideration that data for all three studies above were collected between 2004–2005 while ours were collected in 2010. The prevalence of underweight status for Vietnam as a whole and for HCMC and Hanoi areas have likely decreased over that time period.

We observed higher prevalences of overweight, 14.9% and 29.8%, as defined by WHO standard and Asian-specific definitions, respectively, compared to 6.6% and 16.3% for the 2005 VNAOS [19]. However, the prevalences are similar to those reported for the 2004 urban HCMC residents cohort (WHO standard, 15.4%; Asian-specific, 26.2%) and for Hanoi and adjacent provinces (WHO standard, 12.2%, Asian-specific, 27.5%). The prevalence of being overweight as defined by the WHO standard cut-off in our population and in Vietnam overall remain lower than the 20% to 30% reported for many Southeast Asian countries such as Japan, Malaysia, Philippines, Singapore, Thailand, and South Korea with data collected between 1998 and 2004 [34]. Given the trend towards increasing prevalence of overweight/obesity in low- to middle-income countries, particularly those in Asia [7], prevalence estimates of overweight/obesity for the countries noted above have likely risen.

Obesity by WHO standard remained low at 1.1% for our cohort but increased to 4.0% when the Asian-specific cut-off was applied. These prevalences are higher than the 0.4% and 1.7%, respectively, reported for the 2005 VNAOS, which was collected 5 years earlier. Possibly, our prevalence estimates reflect the continuing trend towards decreasing prevalence of underweight status and increasing prevalence of overweight and obesity in Vietnam overall [19]. Furthermore, our population resides in a city that is undergoing higher than national average economic growth; expansion of urban development; growth of urban areas; and shrinkage of rural areas. Urbanization is coupled with greater influences of globalization and increased availability of fatty and high caloric foods and higher food expenditure per capita [35]. Indeed, our obesity prevalences were more closely aligned with those reported for Hanoi City and adjacent provinces (0.5%, WHO standard; 2.2%, Asian-specific) and HCMC (1.8%, WHO standard; and 6.4%, Asian-specific); both regions have economic growth and development similar to if not greater than that in Danang City . Interestingly, HCMC also showed much higher prevalence of underweight status, 20.4%, compared to 12.4% in Danang and 12.6% in Hanoi. Perhaps, the situation in HCMC – the economic hub of Vietnam – could be attributed to the increasing disparity in wealth and the existence of urban slums.

Similar to the national study and study conducted in Hanoi, patterns in prevalence of body weight status were evident across communes by urbanization. That is, lower underweight and higher overweight/obesity prevalence were associated with greater urbanization. The highest prevalence of underweight status occurred in rural communes at 23.5%. In the urban communes, 43.3% had BMI ≥ 23 kg/m^2^ while 21.3% had a BMI ≥ 25 kg/m^2^. These trends likely reflect continuing national and regional expansion of urbanization, growth of mixed urban–rural areas and reduction of rural populations.

The prevalence of underweight increased with age for both standard WHO and Asian-specific definitions of overweight/obesity prevalence decrease after age 65 years. These are in agreement with results reported in the 2005 national survey. The trend of having lower BMI at older ages is not unusual, even in western nations, as age is affiliated with physical parameters such as frailty [36], and mental conditions such as dementia [37], that are manifested by weight-loss.

The prevalence of overweight was highest for those 35–64 and peaks between 55–64 years of age. For Asian-specific BMI definition, factors associated with overweight/obesity included age, living in urban communes, increased systolic blood pressure, and diabetes. Age was not an associated factor when using the WHO standard definition; however, a reported history of myocarial infarction became highly significant. While income did not remain in the model, urbanization was highly associated with overweight status suggesting that urban living, which provides greater opportunities to access inexpensive processed foods and foods with high refined carbohydrate content, may be the driving force in the rise of overweight/obesity occuring in Danang City and perhaps throughout urban Vietnam. Similar to results found for underweight status, the association of overweight/obesity with health conditions such as diabetes, systolic hypertension, and heart attacks are likely consequences of increased BMI. Knowledge of these associations may provide stimulus for interventions in Danang City and in Vietnam to address the increasing prevalences of overweight/obesity and their impact on the chronic disease burden evident in many other low- and middle-resource countries .

Overall, our study suggests that important chronic conditions such as diabetes, higher systolic blood pressure, and cardiovascular risk factors are significantly and independently associated with being overweight/obese when defined by WHO standard. With the exception of heart attack, these same factors remain importantly related to being overweight/obese as defined by the Asian-specific definition. Although it remains unclear which cut-off points are appropriate for which Asian ethnic groups, what is clear is the need to consider alternative BMI definitions of overweight and obesity as a number of important chronic diseases risk factors appear to remain important at BMI cut-offs lower than the WHO standard definition. Non-communicable diseases such as heart disease, diabetes and cancer are now the leading causes of death in low- to middle-income countries. The WHO reports that non-communicable diseases make up the highest proportion of deaths among both men and women in low- and middle-income countries. Twenty-nine percent of these deaths occur in people under the age of 60 in these countries compared to 13% in high-income countries [38].

Several limitations exist in our study. The cross-sectional design precludes the ability to discern temporality, although it is reasonable to suggest that lifestyles may precede diseases and diseases may result from weight status, an indicator of nutritional status. Second, besides blood pressure, which was objectively measured as part of the study, medical conditions such as arthritis, myocardial infarction, hypercholesterolemia were self-reported. Precise knowledge of personal medical history depends on health care utilization behaviors and interest in one’s own health that are likely to vary according to history with the health care system, education, income, and geographical location of residence. Third, our sample was restricted to participants 35 years and older; therefore, our results are not generalizable to those younger than 35 years of age.

## Conclusions

These results present evidence of the double burden of underweight and overweight/obesity in Danang City, consistent with the patterns found in other large cities in Vietnam that are undergoing similar rapid economic growth, urbanization, and westernization of lifestyle. Prevalence of overweight/obesity significantly increased when using Asian-specific BMI. Factors independently associated with underweight and overweight/obesity status by both WHO standard and Asian-specific definition include age, urbanization, and some modifiable lifestyle behaviors. Further studies are needed to help define ethnic specific BMI cut-offs for Vietnam and to explore strategies to reduce the rising prevalence of overweight and obesity.

## Abbreviations

BMI: Body Mass Index;FEV: Forced Expiratory Volume;HCMC: Ho Chi Minh City;VNAOS: Vietnam National Adult Obesity Survey;WHO: World Health Organization

## Competing interests

The authors declare that they have no competing interests or non-financial competing interests.

## Author’s contributions

ALF was the principle investigator of the main research project and was primarily responsible for the administration and conduct of the study. She led the team in formulating the research questions and producing the survey instrument. She provided training to field workers and in epidemiology and research method to the local researchers. KAL was the project coordinator and primary liaison with the local research team. QVN was the local project coordinator. KAL and QVN participated in formulating the research questions, in producing and translating the survey instrument, and in training field workers. They served as conduits with local authorities regarding study design and procedures. They implemented and supervised the study conduct and visited field sites. TTV developed and managed the study database. TTV and QVN double-entered and checked data for quality control. TGNT provided methodological expertise and conducted data cleaning and data analyses. She also provided training to field workers and in epidemiology and data management to the local researchers. KAL was the primary author of this manuscript. TGNT assisted in writing the manuscript. ALF, QVN, and TTV provided critical revision of the manuscript for intellectual content. All authors read and approved the final manuscript.

## Pre-publication history

The pre-publication history for this paper can be accessed here:

http://www.biomedcentral.com/1471-2458/13/35/prepub
